# Deep-Seated Dental Caries

**Published:** 1853-07

**Authors:** W. H. Dwinelle


### Dental Abstracts.

We propose under this head, to give an abstract from various articles connected with dental science, which we find impracticable to publish in full. In this way we hope to keep more perfectly up with the literature of the profession, and while we may not give our readers all that is desired, yet we hope enough to be of some interest, and perhaps, induce some to extend the number of their periodicals. We do not think that any one periodical can publish all, or half of that which is instructive, and would be of service to the practitioner.

As we make these abstracts, we shall frequently have to use our own language, for the purpose of condensing; hence, we shall, as far as possible, properly credit that which is not in our language.

In the April No. of the Recorder, we have a continuation of W. H. Dwinelle's article, on the treatment of deep seated Dr. dental caries. The subject is, for accuracy, divided as follows:

"Class I—Teeth, the nerves of which are only covered by softened bone, but which, otherwise, so far as can be ascertained, are healthy, or are capable of being restored to health.

Class II—Teeth, whose nerves are by accident, in excavating or otherwise, exposed, were lacerated, but which are healthy, or are capable of being restored to health.."

Class III—Teeth, whose nerves are incapable of restoration, and whose destruction is inevitably required."

Class first and second was treated of, and published in Vol. 6, page 237, of Recorder. Teeth under the first classification rank with the Dr., first in importance, because nearest approaching to nature in her "original condition."

Those under the second classification, second in importance, and third as last. In the article before us, the Doctor first gives several striking cases, showing the recuperative powers of the system, and his change of opinion in relation to this subject—goes on to remark that his "subsequent experience has created an entire revolution in my views upon the subject, as well as course of practice. I am now fully established in the belief, that in a large majority of instances wherein the nerve has been exposed, even to bleeding; the tooth is capable of being restored to all its functional vitality, and to unimpaired and permanent usefulness. I know the profession is, and has been largely, and I doubt not, honestly, divided on this subject. I am happy, however, for the sake of the profession, in believing that confidence is continually increasing in favor of the operation, many who, a few years ago were strong advocates for the removal of the nerve whenever, or however exposed, are now enthusiastic and confident in aiding nature to recuperate herself." Although my method of treating and filling over exposed nerves, may not be essentially different from that already familiar to the readers of the Journal, I trust I shall be excused for introducing it here, if for no other reason that to give consecutiveness and completeness to the treatment of the subject under consideration.

To do this more conveniently, I shall subdivide this class of exposed nerves into two subdivisions.

First, healthy nerves which have been recently exposed in the removal of caries, or by other causes. Secondly, inflammed nerves, which are exposed."

After describing cases when the nerve may be accidentally exposed, and particularly in young persons "whose pulp cavity is comparatively near the surface," he gives the following as the mode of treatment.

"In treating a tooth whose nerve has been lacerated to bleeding, as above, it is my practice not to check its flow at once, but to let it continue until it has nearly exhausted itself, which will generally be within a few minutes from its commencement, then with a lock of cotton saturated with concentrated spirits of camphor, combined with a small quantity of tannin, I wash out the cavity several times; this induces a retraction and collapse of the already exhausted blood vessels, while in my opinion, it often, if not generally, stimulates the formative apparatus of the nerve to make immediate osseous deposit, to repair the violence which has been done."

After speaking of the manner in which ossific matter is deposited to protect the nerve, he goes on to remark: That "after the bleeding of the nerve has once been arrested by the method described, no fear need be entertained of its bleeding after the cavity is plugged. I have never known an instance to occur. The torn blood-vessels seem to retract upon themselves, and heal at once.

Having carefully prepared the cavity for the introduction of the gold, I now construct a concave cap of gold plate, and adapt it to its place at the bottom of the cavity, this is then dropped into a little dish of melted wax, on removing it, it will be found to be very thinly coated over with wax, so thin as scarcely to be perceptible. This give it a non-conducting surface, while the wax about its edges, which come in contact with the bottom of the cavity, will enable you with great certainty, to keep it in its place. The cavity being thoroughly dried, the cap is arched over the nerve, several rings of foil are first introduced and consolidated about the edges of the cap, when the operation is completed in the usual manner. Teeth whose nerves are exposed, but do not bleed, are treated in precisely the same manner, with the difference, of course, that the operation is not retarded by bleeding."

This treatment of Dr. Dwinells differs a little from ours, and we now point out that difference. Instead of using the concentrated spirits of camphor, we use, for the same object, creosote and tannin, formerly collodion—the latter, however, on first application gives more or less pain, and for this cause has been to some extent laid aside. The creosote is unpleasant, and we have no doubt the camphor answers full as well; we have thought it might at first give the same pain which the collodion does. This, however, as a stimulant to excite the deposite of osseous matter, may be requiste. For the last two or three years we but seldom make a concave arch of gold plate; but our plan is more similar to that pursued by Dr. Harris, and to which Dr. Dwinelle alludes, forming an arch with the foil in plugging. While we wish to avoid pressure on the nerve, we also wish to avoid space between the nerve and gold; we wish this space to be as small as possible. The nerve is generally found in the most depending portion of the cavity, and has retracted by the use of tannin and creo-sote or camphor. We place over this, as a non-conductor, a small piece of oil silk, merely so large as that it may rest on the solid bone around the dental foramen. If the cavity is deep, we fold a strip of gold some ten or twelve thickness of foil, and large enough to cover the bottom of the cavity. This is placed over the nerve, the edges compressed or pressed down around the nerve, without pressure over the ne rve itself we then set in a few blocks of foil around the cavity, leavin that portion where we finish, or anterior to us unfilled, consolidating to the walls of the cavity pretty firmly as we proceed, and over the nerve itself set one or two blocks, and before these are forced down we force in at the anterior portion of the cavity, or where we finish the introduction of the gold, as much foil as we can, this holds in place the central blocks, and if the operation has been thorough, we can make almost any amount of pressure without pain. In small cavities we sometimes use but two or three small blocks, which are so arranged as to hold the fold of gold over the nerve in place, and then finish by the introduction of the gold with a small pointed filler, the thickness and size of the folds of gold laid over the nerve should be in proportiont o the size and depth of cavity.

Our success has been far greater than we ever anticipated; but we would not suppose any better, if as good as Dr. Dwinelle's. We like the manner he adopts of getting a non-conducting substance over the nerve, and his whole treatment we think is based on correct pathological principles. But we now give the Dr's. treatment of inflammed nerves, he remarks that "we now come to the consideration of inflamed nerves, which are exposed, but where inflammation has not proceeded so far as to produce disorganizing results; premising, also, that they have been recently exposed. If the inflamed nerve proves to be considerably congested, and is so situated that it can be distinctly seen, and readily approached with an instrument, guided by a careful hand; I have often found it advantageous, if it has not already been done, to carefully rupture its membrane, so as to induce bleeding. This cannot always be done, owing to its situation, or the extreme sensitiveness of the patient, but when the engorged vessels are relieved by this depletion, I have found the nerve much more yielding to subsequent treatment. Tannin, spirts of camphor, and tannate of lead, for applications within the teeth, will be found of great value, while lancing about the neck of the teeth, together with the use of cathartics for the general system, will usually unite in soon reducing the inflamed organ to a condition of comparative health, when it may be capped and filled by the method already described."

Should inflammation after this supervene, the Dr. recommends the removal of the plug, destruction of the nerve and filling of the nerve cavity to the extremity of the fang. The articles from Dr. Dwinelle on the treatment of exposed nerves, are worth a careful perusal, and we are satisfied that if more of the profession were to adopt his practice, and pursue it faithfully, many teeth now lost would be saved.

